# Response of tef yield and yield components to nitrogen and phosphorus fertilizers

**DOI:** 10.1371/journal.pone.0299861

**Published:** 2024-03-19

**Authors:** Ewunetie Melak, Workat Sebnie, Tilahun Esubalew, Haymanot Lamesgn, Messay Abera, Tesfa Asmelie

**Affiliations:** Soil and Water Research Directorate, Sekota Dryland Agricultural Research Center, Sekota, Ethiopia; United Arab Emirates University, UNITED ARAB EMIRATES

## Abstract

The challenge facing Ethiopian farmers are the combination of low soil fertility and reduced agricultural productivity. The study aimed to quantify nitrogen and phosphorus-containing fertilizers for tef production in Sekota and Lasta-Lalibela districts, using four nitrogen and three phosphorus levels from urea and triple super phosphate sources. The Results showed that nitrogen and phosphorus fertilizers significantly affected tef yield and yield components. Application of nitrogen at the rate of 92 kg ha^-1^ increased tef grain yield by 131.01%, 87.78% and 182.23% in Woleh, Sayda and Lasta Lalibela districts, respectively, compared to control treatment. Similarly, 23 kg ha^-1^ phosphorus increased tef grain yield by 7.42 and 8.52% in Woleh and Lasta Lalibela, respectively. In Lalibela and Woleh, the application of 92 kg N ha^-1^ resulted in a maximum net benefits of 2099.6 and 2007 United States Dollar (USD) respectively. Furthermore, in Sayda, the application of 92 kg N ha^-1^ and 23 kg P_2_O_5_ ha^-1^ yielded a net benefit of 1812.55 USD. The marginal rate of return (MRR) from these applications was 539.6% in Lalibela, 781% in Woleh and 352.44% in Sayda. Therefore, application of 92 N and 23 kg P_2_O_5_ ha^-1^ is appropriate for maximum tef production in Sekota and Lasta districts of Amhara region. Further research and extension services should focus on promoting the adoption of these optimized fertilizer practices among smallholder farmers to maximize the sustainable production of tef.

## Introduction

Tef (*Eragrostic tef (Zucc*.*)* Trotter) is the main indigenous cereal crop of Ethiopia, where it was originated and diversified [1]. It is a highly demanded cereal and a staple food grain for more than 85% of the 110 million people of Ethiopia. It has higher market prices in both (grain and straw) than the other cereals [2]. Due to its gluten free nature and other merits, the current acceptance of tef in Europe, USA and other regions of the world is increasing. Studies confirm that tef is a healthy, reliable and low risk crop [3]. Similarly, Spaenij et al. [4] reported that the absence of gluten in its grain makes tef a healthy food such that people allergic to gluten can safely consume tef products. The price is increased over the last decade from 62.5 to 312.5 USD per ton [2]. Currently, its price is more than 1718.75 USD per ton and linearly increasing.

Nitrogen deficiency is prevalent in almost all soils, and phosphorus deficiency occurs in 70% of Ethiopian soils [5]. Regardless of soil type, agroecology, landscape, or geographic region, nitrogen has consistently emerged as a major nutrient limiting crop yield [6, 7], and researchers such as Hirel et al. [8] have also identified nitrogen as a common factor limiting yield. Nutrient losses (including NPK) are high in the study area due to undulating land, intense rainfall, poor fertilizer retention in crop residues, and absence of external nutrient sources [9].This problem is not limited to the study area, but is widespread throughout Ethiopia. As a result, Ethiopia imports large quantities of fertilizers containing nitrogen, phosphorus, sulfur, zinc, and boron [10]. Synthetic nitrogen fertilizers contribute to a 50% increase in global food crop yields [11].

Various studies conducted in different districts of Ethiopia have demonstrated the positive effects of nitrogen and phosphorus fertilization on tef yield and growth parameters. For instance, Dereje et al. [12] found that application of NP fertilizer at a rate of 46/23 in the Assosa area increased grain yield by 137%. Similarly, Fissehaye et al. [13] and Yared et al. [14] reported increased grain yield in the Guji Zone and Tigray areas, respectively, when using an NP ratio of 69/69. Tamirat [15] conducted a study in southern Ethiopia and discovered that applying 97.5 kg N ha^-1^, combined with an intra-row spacing of 25 cm, resulted in a grain yield increase of 1713.3 kg ha^-1^. These studies highlight the importance of nitrogen and phosphorus fertilizers for tef production and productivity. They confirm the importance of site-specific nutrient management for tef production. Soil fertility conditions vary dynamically from region to region, making it difficult to offer one recommendation for different locations. It is crucial to implement site-specific nutrient management strategies to enhance crop production. Despite the importance of tef, as mentioned before, tef productivity remains low. This is due to the limited research conducted on determining appropriate NP fertilizer rates in the study areas. The lack of research has impeded the optimization of tef productivity through targeted fertilization, forcing farmers to rely on traditional (blanket recommendation) and often inefficient fertilization methods. Consequently, the average yield of tef in the study areas does not exceed 1.1 t ha^-1^ [16]. Therefore, the objective of this study was to investigate the effects of nitrogen and phosphorus fertilizers on tef yield and yield components in the Wag-Lasta areas.

## Materials and methods

### Description of the study area

**Fig 1:**
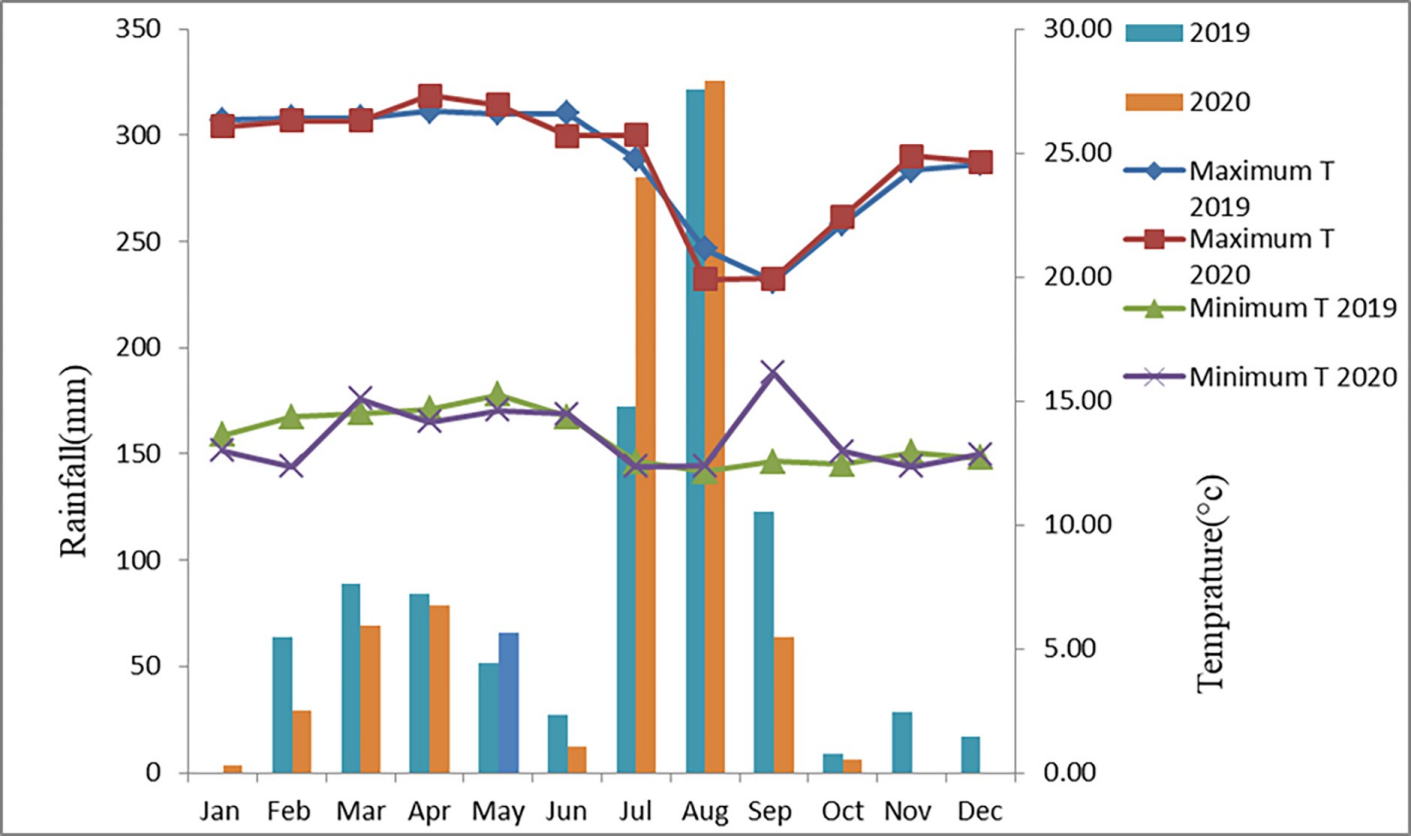
The annual rainfall and maximum and minimum temperature of Lasta Lalibela district.

**Fig 2:**
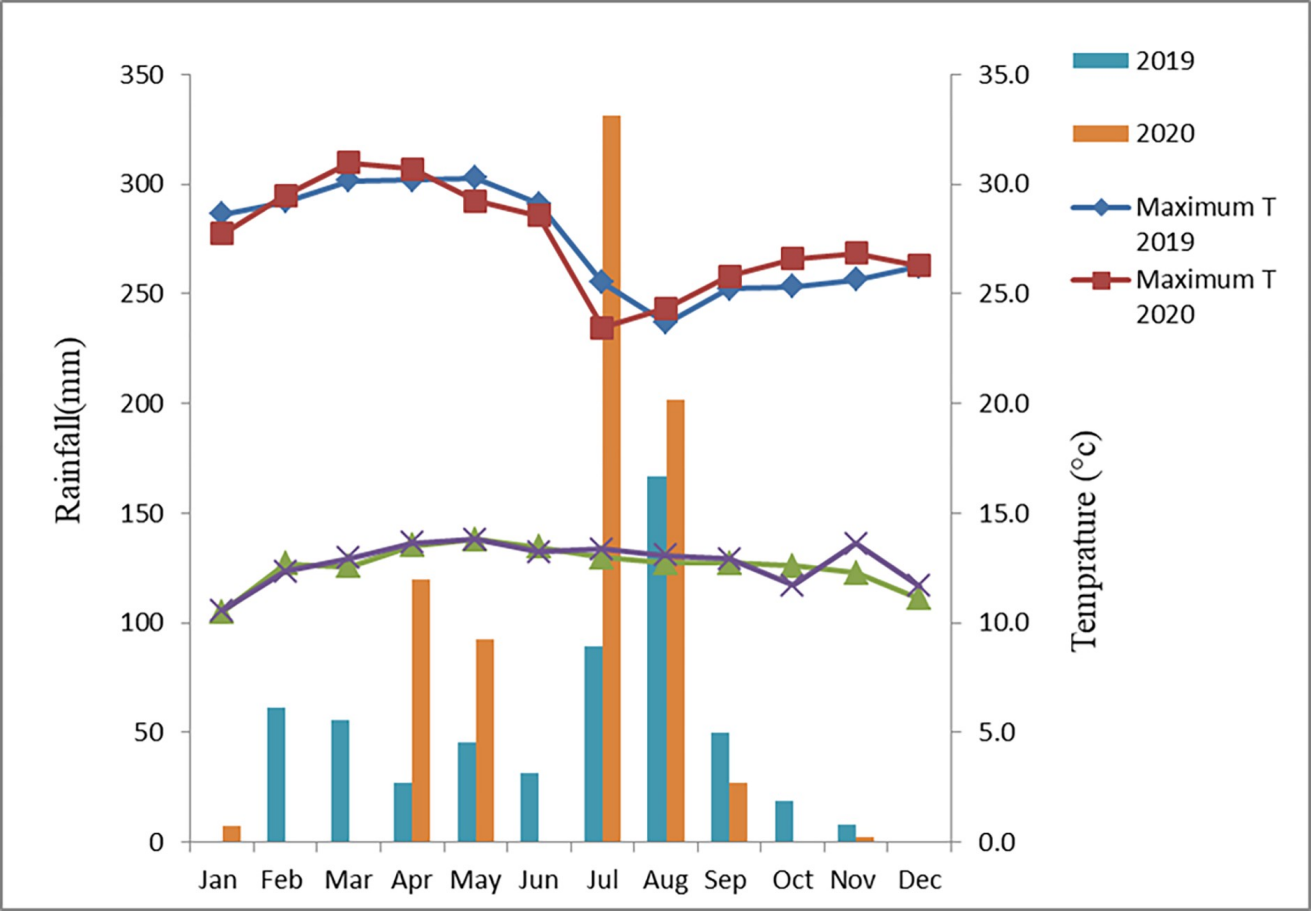
The annual rainfall and maximum and minimum temperature of Sekota district.

The experiment was conducted at Sekota and Lasta districts of eastern part of Amhara Region, Ethiopia. The districts are located in Wag-himra and north Wollo zone of Amhara regional State. These areas are covered by undulated topography, uneven distribution and erratic rain fall, very shallow soil depth, high soil erosion, bad management practice and scattered forest coverage. Soil erosion by water and low productivity are a serious problem in the study areas [9, 17]. The major crops which are grown in these districts are: sorghum, tef, wheat, barley, maize, and fababean & check pea. The experiment was conducted during the main cropping season of rainfed agriculture without irrigation. There were no irrigation facilities in the area, and the crop depended solely on rainfall. However, irrigation was practiced in neighboring areas where irrigation facilities and surface water was readily available. The agro-ecology of the experimental sites is classified as midland [18].The annual rainfall of Sekota area is about 589.2 mm and the average minimum and maximum air temperatures are 12.7 and 27.4°C, respectively, whereas the annual rainfall in the Lasta-Lalibela area is about 799.3 mm and the mean minimum and maximum air temperatures are 13.5 and 24.7°C, respectively (Kombolcha meteorological station). Annual rainfall and maximum and minimum air temperatures, recorded at Lasta Lalibela and Sekota districts during the 2019 and 2020 cropping seasons are shown in Figs 1 and 2, respectively (S1 and S2 Files).

### Treatments and experimental design

The experiment was conducted in twelve farmers’ fields for two consecutive years (2019–2020) at four Kebeles (local administrative subdivisions) located in two different districts: Nakutolab and Medagie in Lasta Lalibela district, and Woleh and Sayda in Sekota district. Experiments and experimental sites were approved by Amhara Agricultural Research Institute and Sekota Dryland Agricultural Research Center. The treatments in the experiment comprised a factorial combination of four levels of N (0, 46, 69, and 92 kg N ha^−1^) and three levels of P_2_O_5_ (0, 23, and 46 ha^−1^), resulting in a total of 12 treatments. The experiment followed a randomized complete block design (RCBD) and was replicated three times. The experimental plots were sized at 3 m by 3 m, providing a net harvestable plot area of 7.8 m^2^. Because, two rows per plot were excluded as boarder effect.

The source of N and P fertilizers were urea (NH_4_)_2_CO_3_ and triple super phosphate (TSP), respectively. Application of urea was in two splits, half at planting and half at vegetative, while the full rate of phosphorus was added during sowing. Q*uncho tef* variety was used as a test crop with the recommended seed rate of 10 kg ha^-1^. The spacing between block, plot and row were 1 m, 0.5 m and 0.2 m, respectively. The experimental sites were adequately prepared to a depth of 0–20 cm using local *maresha* plows pulled by oxen. Manual planting of tef was carried out on the well-prepared sites. Throughout the growth period of tef, manual weeding (hand weeding) practices were conducted three times. The experiment was harvested by using sickle and threshing activity also performed manually by labor.

### Soil sampling and analysis method

Prior to planting, composite soil samples were collected using an auger from the 0–20 cm depth. The composite samples were sampled by combining 20 subsamples taken from each experimental site using a zigzag sampling method. The collected soil samples were air-dried, ground, and sieved using 0.5 mm and 2 mm sieves. The collected and prepared samples were analyzed for texture, pH, organic carbon, total nitrogen, available P, and electrical conductivity (EC) following the standard procedure in Sekota Dryland Agricultural Research Center Soil Laboratory. The determination of particle-size distribution was done by using the hydrometer procedure method [19]. Soil organic carbon was evaluated using the wet digestion method [20].Total nitrogen content was analyzed using the micro Kjeldahl method [21]. Soil pH was measured using a digital pH meter [22] with a soil to water ratio of 1:2.5. Available phosphorus content was determined using the Olsen method [23].

### Soil properties of experimental sites before sowing

Laboratory results indicate that the pH of the experimental soil ranged from 6.2 to 7.25, which is slightly acidic to neutral. This soil pH suggests that, it is favorable for plant growth, and fertilizer application is crucial to raising the yield of tef [24]. The amounts of soil organic carbon were in between 0.039% to 0.6825%, it indicates that the OC was very low and low range. Soil total nitrogen content ranged from 0.0042% to 0.0308%, very low [25]. The results showed that to significantly increase of yields, external inputs such as organic and inorganic fertilizers must be added. Without these inputs, soil levels of organic carbon (OC) and total nitrogen (N) alone were insufficient to significantly increase crop productivity.

The available phosphorus content of the soils fell into the high category, ranging from 14 to 75 mg kg^-1^ [23]. This indicates that the majority of the soils are rich in phosphorus and that the application of phosphorus-containing fertilizers may be unresponsive and uneconomical. Soil conductivity also ranged from 0.03 to 0.13 dS/m (deci-siemen-per-meter) at all experimental sites, classifying them as normal soils without salts [26] (Table 1). In general, soil analysis results indicated that soil nutrients, except phosphorus, were deficient and needed to support potential crop production. This could be related to high soil erosion, poor cropland management, and continuous cropping with complete removal of crop residues, which led to reduced soil fertility.

**Table 1:** Soil properties of experimental sites at planting time.

Soil parameter	Site 1	Site 2	Site 3	Site 4	Site 5	Site 6	Site 7	Site 8
pH	6.9	6.8	7.25	6.7	6.2	6.2	6.6	6.6
%OC	0.35	0.37	0.04	0.68	0.62	0.66	0.59	0.68
%TN	0.01	0.01	0.01	0.02	0.03	0.01	0.02	0.03
P (mg kg^-1^)	75	45.6	22	14	66	44.78	25.1	21
EC (dS/m)	0.03	0.03	0.03	0.16	0.13	0.12	0.13	0.11

%OC = organic carbon in percent, % TN = total nitrogen in percent, P (mg kg^-1^) = available phosphorus in milligram per kilogram soil, EC = electrical conductivity of soil in desi siemens per meter.

### Data collection

The collected agronomic data were plant height, panicle length, biomass and grain yield. The plant height and panicle length (m) parameters were collected from the five randomly selected samples, while both aboveground biomass and grain yield (kg ha^-1^) data were collected from the net harvestable experimental units. The plant height of five (5) randomly selected plants was measured at physiological maturity, specifically from the base of the stem to the tip of the plant by using measuring tape. Similarly, the panicle length was identified by measuring the average length of the main tiller panicles from five (5) randomly selected plants at physiological maturity. Determination of aboveground biomass was involved by weighing the total air-dried biomass harvested from net plot areas and converted to kg ha^-1^. The grain yield also determined by measuring the weight of the grains from plants within a net plot area at harvest maturity and converted to kg ha^-1^.

### Partial budget analysis

The actual grain yield was adjusted downward by 10% to equalize the experimental yields with what farmers would expect to get from the same treatment. The costs that varied among all the treatments were fertilizer purchasing cost by considering the current price [27]. The economic analysis was done based on the formula established by CIMMYT [28]. Treatment dominance analyses were carried out first by listing the treatments in order of increasing cost variation to identify the economically preferable treatments.

### Data analysis

The effects of N and P-containing fertilizers on yield and yield components of tef were statistically tested. The analysis of variance (ANOVA) was performed to assess the difference between treatments using R software (R 4.2.2: R core team (2022) R: The R Foundation for Statistical Computing, Vienna, Austria). Whenever treatment effects were significant, the mean separation was done by least significance difference (LSD) at the p < 0.05 level of significance.

## Results and discussion

### ANOVA mean squares for the impacts of nitrogen and phosphorus on the plant height, panicle length, biomass, and grain yield of tef

In all locations, the application of nitrogen fertilizer had a significant impact (P ≤ 0.001) on various parameters of tef, including plant height, panicle length, above-ground biomass, and grain yield. Phosphorus did not have a significant effect on most parameters, except for a plant height at Lasta-Lalibela and grain yield at Sayda sites. These findings suggest that the response of tef to nitrogen and phosphorus fertilizers may vary by location. Significant effects on plant height, panicle length, above-ground biomass, and grain yield indicate the importance of nitrogen fertilizer in promoting overall growth and productivity of tef. However, the limited impact of phosphorus fertilizer on most parameters implies that its role may be more specific and location-dependent. However, in Sekota (Woleh, Sayda) and Lasta-Lalibela districts, the interaction of N and P fertilizer had no effect on plant height, panicle length, biomass and grain yield, while in Sayda, the interaction a significant effect on grain yield (Table 2). This suggests that the combined application of nitrogen and phosphorus fertilizers had a synergistic effect on crop productivity in Sayda.

**Table 2:** The effect of nitrogen and phosphorus on the growth parameter of tef at Woleh Sayda and Lasta-Lalibela.

Mean square values					
Source of variation	DF	Woleh			
		PH(m)	PL(m)	BY (kg ha^-1^)	GY (kg ha^-1^)
N	3	0.2059***	0.0265***	319207***	2265610***
P	2	0.0054^ns^	0.0046^ns^	265134^ns^	71507^ns^
N*P	6	0.0038^ns^	0.0009^ns^	384667^ns^	81699^ns^
Error	22	0.0088	0.0017	640521	101867
Sayda					
N	3	0.064***	0.0064*	235907***	1316942***
P	2	0.0087^ns^	0.0003^ns^	540683^ns^	463290***
N*P	6	0.0017^ns^	0.0009^ns^	323320^ns^	116686*
Error	22	0.0096	0.0017	321328	37507
Lalibella					
N	3	1.2189**	0.0977***	1686708**	1513407***
P	2	0.0291**	0.0011^ns^	919789^ns^	423799*
N*P	6	0.0043^ns^	0.0003^ns^	1208271^ns^	76115.7^ns^
Error	22	0.0052	0.0008	1161602	116930

PH plant height; PL panicle length; BM biomass; GY grain yield; N nitrogen; P phosphorus *Significant at 0.05 probability level; **Significant at P < 0.01 probability level. Ns non-significant at 0.05 probability level

### Effect of nitrogen and phosphorus containing fertilizers on shoot parameters and yield component of tef

The shoot parameter (plant height) and yield component (panicle length) of tef were linearly increased with increasing rate of nitrogen at all locations. The plants that received higher levels of nitrogen fertilizer showed a greater increase in both plant height and panicle length compared to those that received lower levels. This suggests that nitrogen plays a crucial role in promoting overall plant growth and development (Table 2). The application of 92 kg ha^-1^ N fertilizer increased plant height by 26.4%, 16.5% and 34.2% in Woleh, Sayda and Lasta-Lalibela, respectively compared to control treatment (Table 3). Similarly, panicle length increased by 22.9, 11.8 and 22.2% in Woleh, Sayda and Lasta-Lalibela respectively, compared to control (S3 File).

**Table 3:** Effect of nitrogen and phosphorus for plant height and panicle length of tef at Woleh, Sayda and Lasta district.

N (kg ha^-1^)	Sekota district				Lasta district	
	Woleh		Sayda			
	PH (m)	PL (m)	PH (m)	PL (m)	PH (m)	PL (m)
0	0.86	0.35	0.79	0.34	0.79	0.36
46	1.07	0.42	0.90	0.38	1.01	0.42
69	1.06	0.42	0.92	0.38	1.05	0.43
92	1.09	0.43	0.92	0.38	1.06	0.44
LSD	0.063	0.03	0.07	0.03	0.02	0.01
P_2_O_5_ (kg ha^-1^)						
0	1.01	0.397	0.87	0.37	0.96	0.41
23	1.00	0.399	0.88	0.37	0.99	0.41
46	1.03	0.423	0.91	0.37	0.99	0.41
LSD	Ns	0.02	Ns	Ns	0.02	Ns
CV%	9.23	10.02	7.87	10.3	7.38	6.99

LSD = least significant difference; CV% = coefficient of variation in percent.

Maximum plant height and panicle were recorded at a rate of 92 kg ha^-1^ but, in statistical parity with application rates of 69 and 46 kg ha^-1^ at Woleh and Sayda, respectively. In Lasta-Lalibela district the highest growths were registered by the application of nitrogen at a level of 92 kg ha^-1^ but, similar to the application rate of 69 kg ha^-1^. The lowest growth parameters were recorded at the control treatment. This increase in plant height and panicle length was probably due to nitrogen fertilizer stimulates the vegetative growth of plant. The stimulation of vegetative growth by nitrogen fertilizer can lead to several beneficial effects. Firstly, increased plant height allows for better light interception and photosynthetic activity, which can enhance the overall productivity of the plant. Secondly, longer panicles provide a greater surface area for flower and grain development, potentially leading to increased yield [29]. Studies in different districts of Ethiopia have confirmed the positive impact nitrogen fertilizer levels on growth parameters of tef. The findings of this study are align with previous studies by [15, 30], who reported that increasing nitrogen fertilizer application leads to substantial improvements in important yield attributes, such as plant height and panicle length. Additionally, these results are consistent with the findings reported by [12–14, 31]. They observed that increasing nitrogen fertilizer rates positively influenced the growth parameters of tef, specifically plant height and panicle length. Similarly, maximum N fertilizer application to wheat crop resulted in higher plant height compared to the control (no fertilizer) treatments [32, 33].

The maximum plant height and panicle length from P fertilizer was recorded from the application of 46 kg ha^-1^P_2_O_5_ but statistically par with the application of 23 P_2_O_5_ kg ha^-1^ and the control treatment. This might be the inherent soil P level enough for plant and panicle length development of tef, since its level is above the critical range.

### Effects of nitrogen and phosphorus fertilizer on grain yield of tef

The highest grain yields were observed in plots treated with the highest nitrogen fertilizer rates, indicating that nitrogen had positive effect on tef productivity in all locations. But, phosphorus significantly influenced the grain yield only at Sayda and Lasta-Lalibela (Table 2). The maximum grain yields obtained across all sites were more than double compared to the control treatment (Table 4, S3 File).

**Table 4:** Effect of nitrogen and phosphorus on grain yield of tef.

	Woleh	Lasta-Lalibela
N kg ha^-1^	GY (kg ha^-1^)	GY (kg ha^-1^)
0	925.9	785.8
46	1532.8	1434.7
69	1564.2	1688.7
92	1738.7	1815.3
LSD	218.9	112.2
P_2_O_5_ kg ha^-1^		
0	1378.3	1356.0
23	1462.3	1482.3
46	1480.6	1455.0
LSD	212.9	97.2
CV	22.16	23.89

This substantial improvement in yield can be attributed to the nutrient-deficient status of the soil, which was found to be below the critical level. The grain yield of tef increased as the rates of nitrogen increased from 0 to 92 kg ha^-1^. But the application of 92 kg ha^-1^ nitrogen was not statistically significant with applied rates of 46 and 69 kg N ha^-1^. In Sekota (Woleh, Sayda) and Lasta-Lalibela districts, the application of 92 kg ha^-1^ nitrogen increased yields by 131.01%, 87.78%, and 182.23%, respectively, compared to the control treatment. Similarly, yields obtained from this study were higher than the national recommendations. This suggests that the N fertilizer used in the study were effective in maximizing tef yields. These findings have important implications for farmers and agronomists, as they demonstrate the potential for surpassing national standards and achieving higher yields with the right approach.

The current result is consistent with the finding of Gebretsadik et al. [30], who reported that the application of nitrogen at a rate of 92 kg ha^-1^ significantly increases the grain yield of tef. Similarly, Tamirat et al. [15] reported that the mean grain yield of tef was significantly affected by the application of nitrogen at a rate of 97.5 kg N ha^-1^. Research ascertained by Tekalign et al. [5] and Minale et al. [34] showed that an increasing amount of nitrogen increases the grain yield of tef. This increment of yield by nitrogen might be due to the fact that it is an essential nutrient and component of the chlorophyll molecule. Increased nitrogen level has been observed to positively impact the leaf chlorophyll content. Higher nitrogen levels increase chlorophyll content, which in turn increases seed yield [29].

Likewise, phosphorus also had a significantly effect on tef grain yield in Lasta-Lalibela. The application of phosphorus increased the grain yield by 8.52% (Table 4). In both locations, the yield from nitrogen application was higher than from phosphorus application. This suggests that nitrogen is the primary limiting nutrient in the study sites, as nitrogen application had a more significant impact on yield compared to phosphorus. Amare et al. [35] provided supporting evidence that nitrogen fertilizer is the primary and most significant yield-limiting nutrient, followed by phosphorus, confirming the idea that the decline in soil fertility affects crop production. Likewise, Alemayehu et al. [36] and Chala et al. [7] reported similar findings in their studies. The omission of nitrogen (N) and phosphorus (P) had a highly significant impact on reducing both the grain yield and yield components of tef.

The maximum grain yield (1640.6 kg ha^-1^) was obtained from the application rate of 92 kg N and 46 P_2_O_5_ kg ha^-1^ at Sayda. While the minimum yield (581.3 kg ha^-1^) was obtained from unfertilized treatment. The yield was increased with increasing rate of nitrogen and phosphorus fertilizer but statistically par with rates of 92/23, 69/46, 46/46 kg ha^-1^ N and P respectively (Table 5). Currently, phosphorus (P) is not a limiting factor for tef production in Lasta-Lalibela and Woleh. However, if no P is applied at all, it could potentially become a limiting nutrient in the future. Therefore, it is crucial to apply a minimum rate of 23 kg P_2_O_5_ ha^-1^ to ensure the maintenance of adequate soil P levels.

**Table 5:** Interaction effects of N and P fertilizer on yield of tef at Sayda.

N kg ha^-1^	P kg ha^-1^		
	0	23	46
0	581.3	1162.6	1094.8
46	1363.8	1475.2	1510.3
69	1428.9	1377.1	1575.6
92	1422.9	1598.1	1640.6
LSD	223.8		
CV	22.16		

### Effect of nitrogen and phosphorus on biomass yield of tef

Application of nitrogen increased the biomass yield of tef by 67.18%, 103% and 131.69% in Sekota (Woleh, Sayda) and Lasta-Lalibela districts, respectively, over control treatment (S3 File). In all experimental sites, biomass yield tended to increase with increasing nitrogen rates. Without nitrogen fertilizer, biomass yield decrease to twice that of the control treatment. The low nitrogen concentration of the soil study sites may be the cause of this, as nitrogen is essential for photosynthesis and chlorophyll, which increase tef’s biomass [37, 38].

However, the application of phosphorus fertilizer did not have a significant impact on the biomass yield of tef across all sites. There were numerical differences observed among the treatments with varying phosphorus levels (Table 6). This could be attributed to the fact that the inherent soil phosphorus status was already above the critical value (Table 1). These findings are consistent with the research conducted by Haftamu et al. [30] and Temesgen [39], which also demonstrated that higher rates of nitrogen application resulted in the highest biomass yield. This can be attributed to factors such as increased panicle size and plant height, which are enhanced by nitrogen-containing fertilizers, ultimately leading to greater biomass yield and straw yield. Adissie et al. [40] also verified that the higher (92 kg ha^-1^ N) rate produced the highest biomass yield of tef.

**Table 6:** Effect of nitrogen and phosphorus fertilizers on biomass yield of tef.

N (kg ha^-1^)	Sekota district		Lasta-Lalibela
	Woleh	Sayda	
	BY (kg ha^-1^)	BY (kg ha^-1^)	BY (kg ha^-1^)
0	2919.2	3881.3	2666.6
46	5106.1	5805.7	4841.2
69	5451.3	5997	5557.0
92	5929.1	6488.7	6178.5
LSD	534.01	378.23	353.70
P_2_O_5_ (kg ha^-1^)			
0	4736.6	5406.1	4698.9
23	4874.5	5519.9	4880.5
46	4942.9	5703.5	4853.1
LSD	Ns	Ns	Ns
CV	16.50	21.79	22.40

### Partial budget analysis

According to the partial budget analysis, the highest net benefits were observed when nitrogen fertilizer was applied in Lasta-Lalibela and Woleh, and when both nitrogen and phosphorus fertilizers were applied in Sayda. The maximum net benefits amounted to 2099.63 USD in Lasta-Lalibela, 2006.99 USD in Woleh, and 1839.54 USD in Sayda. These results were obtained from applying 92 kg ha^-1^ of nitrogen in Lasta-Lalibela and Woleh, and 92N X 23P_2_O_5_ kg ha^-1^ in Sayda, with corresponding marginal rate of return (MRR) values of 539.61%, 781.00%, and 110.60% (Tables 7–9).

**Table 7:** Partial budget analysis at Lasta-Lalibela.

Treatments	UY (kg ha^-1^)	AY (kg ha^-1^)	GB(USD)	VC (USD)	NB(USD)	MRR%
N (kg ha^-1^)						
0	785.8	707.22	950.3	0.0	950.3	-
46	1434.7	1291.23	1735.1	47.9	1687.2	1539.19
69	1688.7	1519.83	2042.3	71.8	1970.5	1183.26
92	1815.3	1633.77	2195.4	95.8	2099.6	539.61
P_2_O_5_ (kg ha^-1^)						
0	1356	1220.4	1639.9	0.0	1639.9	-
23	1482.3	1334.07	1792.7	24.4	1768.3	525.84
46	1455	1309.5	1759.6	48.8	1710.8	D

ETB = Ethiopian birr, UY = Unadjusted, AY = Adjusted yield, GB = Gross benefit, VC = variable cost, NB = Net benefit

**Table 8:** Partial budget analysis at Woleh.

Treatment	UY(kg ha^-1^)	AY (kg ha^-1^)	GB (USD)	VC (USD)	NB (USD)	MRR%
N (kg ha^-1^)						
0	925.9	833.31	1119.76	0	1119.76	
46	1532.8	1379.52	1853.73	47.88	1805.86	1433
69	1564.2	1407.78	1891.70	71.81	1819.89	59
92	1738.7	1564.83	2102.8	95.75	2007.00	781
P_2_O_5_ (kg ha^-1^)						
0	1378.3	1240.5	1666.88	0	1666.88	
23	1462.3	1316.1	1768.47	24.41	1744.06	316
46	1480.6	1332.5	1790.60	48.81	1741.78	D

**Table 9:** Partial budget analysis at Sayda.

N (kg ha-^1^)	P_2_O_5_(kg ha-^1^)	UNADY(kg ha^-1^)	ADY (kg ha^-1^)	Gross benefit (USD)	Variable Costs (USD)	Net benefit (USD)	MRR%
0	0	581.3	523.17	703.01	0	703.01	
0	23	1162.6	1046.34	1406.02	24.41	1381.61	2780.3
46	0	1363.8	1227.42	1649.34	47.88	1601.47	936.8
0	46	1094.8	985.32	1324.02	48.81	1275.21	D
69	0	1428.9	1286.01	1728.08	71.81	1656.26	228.9
46	23	1475.2	1327.68	1784.07	72.28	1711.79	11845.4
92	0	1422.9	1280.61	1720.82	95.75	1625.07	D
69	23	1377.1	1239.39	1665.43	96.22	1569.21	D
46	46	1510.3	1359.27	1826.52	96.69	1729.83	73.93
92	23	1598.1	1438.29	1932.70	120.15	1812.55	352.44
69	46	1575.6	1418.04	1905.49	120.63	1784.87	D
92	46	1640.6	1476.54	1984.10	144.56	1839.54	110.6

UNADY = unadjusted yield, ADY = adjusted yield, MRR = marginal rate of return, USD = United States Dollar

## Conclusion and recommendation

In the northern regions of Ethiopia, particularly in the Wag-Lasta areas, nitrogen deficiency is a prevalent issue in the soils. This study emphasizes that the application of nitrogen across all sites, along with phosphorus in specific locations, can significantly improve both the overall yield and yield components of tef. The research findings unequivocally identify nitrogen as the most critical nutrient limiting yield in the study areas. Consequently, the recommended fertilizer application rate for optimizing tef production in the Sekota and Lasta districts of the Wag-Lasta area is 92 kg ha^-1^ of nitrogen. Additionally, it is recommended to apply 23 kg ha^-1^ of P_2_O_5_ in all sites as a means of maintaining soil phosphorus levels. Adhering to these recommended practices has the potential to double the yield, making a substantial contribution to addressing food security challenges. Future research initiatives and extension services should prioritize the promotion and adoption of these optimized fertilizer practices among smallholder farmers, with the goal of maximizing the sustainable production of tef.

## Supporting information

S1 File (XLS)

S2 File (XLS)

S3 File (XLS)
